# Role of Reactive Oxygen Species in Pathogenesis of Radiocontrast-Induced Nephropathy

**DOI:** 10.1155/2013/868321

**Published:** 2013-12-29

**Authors:** Antonio Pisani, Eleonora Riccio, Michele Andreucci, Teresa Faga, Michael Ashour, Antonella Di Nuzzi, Aldo Mancini, Massimo Sabbatini

**Affiliations:** ^1^Department of Nephrology, “Federico II” University of Naples, 80131 Naples, Italy; ^2^Department of Health Sciences, “Magna Graecia” University, Catanzaro, Italy; ^3^National Cancer Institute G. Pascale, Naples, Italy

## Abstract

In vitro and in vivo studies have demonstrated enhanced hypoxia and formation of reactive oxygen species (ROS) in the kidney following the administration of iodinated contrast media, which play a relevant role in the development of contrast media-induced nephropathy. Many studies indeed support this possibility, suggesting a protective effect of ROS scavenging or reduced ROS formation with the administration of N-acetylcysteine and bicarbonate infusion, respectively. Furthermore, most risk factors, predisposing to contrast-induced nephropathy, are prone to enhanced renal parenchymal hypoxia and ROS formation. In this review, the association of renal hypoxia and ROS-mediated injury is outlined. Generated during contrast-induced renal parenchymal hypoxia, ROS may exert direct tubular and vascular endothelial injury and might further intensify renal parenchymal hypoxia by virtue of endothelial dysfunction and dysregulation of tubular transport. Preventive strategies conceivably should include inhibition of ROS generation or ROS scavenging.

## 1. Introduction

Contrast media- (CM-) induced nephropathy (CIN) is an acute deterioration of renal function following administration of CM in the absence of any other known reason. CIN remains a leading cause of iatrogenic acute kidney injury, accounting for some 10% of in-hospital acute renal failure, despite adherence to protocols of risk assessment and prevention strategies. This reflects the unremitting increase in radiocontrast procedures for computerized tomography and vascular interventions, especially in high risk and elder patients with major comorbidities [1, 2].

CIN is commonly defined as an increase in serum creatinine concentration >0.5 mg/dL or greater than 25% of its previous value within 3 days after contrast medium administration, in the absence of other different causes [3]. In most cases, after a peak value of renal dysfunction within the 5th day, in which granular casts and a modest proteinuria may appear in urine, plasma creatinine levels return to baseline level by 7 to 10 days; a minority of patients, however, may experience irreversible loss of renal function requiring dialysis and even death [4].

There are several predisposing factors to the development of CIN (Table 1). Preexisting renal failure certainly represents the most common condition associated with CIN, with incidence ranging between 5.3% and 30% according to the different studies and, mostly, to the different degrees of renal failure at time of contrast medium administration and to the total amount of CM employed [1, 5–7]. Indeed, patients with chronic renal failure have defective antioxidant systems [8] and increased oxidative stress associated with inflammation and endothelial dysfunction [9]. A high incidence of CIN is also associated with diabetes (range: 5.7–29.4%); contrary to what is commonly believed, diabetes represents a predisposing factor to CIN only in the presence of chronic renal failure (CRF) since, in patients with preserved renal function, CIN incidence is the same as in nondiabetic patients [10, 11]. It is interesting to note that most of the conditions predisposing to CIN reported in Table 1 are associated with “a priori” increased generation of reactive oxygen species (ROS), that threaten oxygen balance and antioxidant systems.

While there is full agreement about the greater nephrotoxicity of high-osmolarity CM compared to others, it is not clear whether isoosmolar CM are less nephrotoxic than low-osmolarity CM, since clinical studies offer controversial results; all CM, however, may determine acute renal damage. Among procedure-related factors, the amount of injected CM is a crucial factor in determining CIN, since ROS generation appears to be strictly proportional to it [12].

## 2. Physiopathology of CIN: Role of Reactive Oxygen Species

CIN is the result of combined hypoxic and toxic renal parenchymal injury; the latter is presumably mediated by reactive oxygen species (ROS). Experimental findings in vitro and in vivo illustrate that renal hypoxia induced by administration of iodinated CM enhances ROS formation within the kidney. Moreover, the protective effect on renal function of ROS scavengers or reduced ROS formation with specific drugs provides indirect evidence that ROS might be involved in the pathogenesis of CIN.

Under physiological conditions, tubular transport is associated with ROS formation, mostly in the renal medullary thick ascending limb, where the extremely dense mitochondrial population represents a major source for generation of superoxide anions (O_2_^−^) and hydroxyl radicals (OH^−^) by NAD(P)H-oxidase [2]. ROS participate in cellular signaling processes and modulate the actions of various messengers and major transcription factors. At medullary tubular level, ROS generation also plays an important role in regulating microcirculation, through its effects on nitric oxide (NO) levels.

Administration of CM markedly decreases renal oxygenation in medullary structures, without reducing tubular reabsorption, as the result of neurohumoral vasoconstrictive stimuli, following the release of prostaglandins and endothelin from endothelial cells exposed to CM. This latter agent is markedly enhanced following radiocontrast studies, due to the activation of endothelin-converting enzyme-1.

This results in altered oxygen balance, leading to depressed activity of mitochondrial scavengers and enhanced formation of ROS. During hypoxia, greater amounts of ATP are hydrolyzed to ADP and AMP, further metabolized to adenosine and inosine by 5′-nucleotidase, and subsequently to hypoxanthine that generates xanthine and hydrogen peroxide (H_2_O_2_), via xanthine oxidase. The same enzyme also helps xanthine generate uric acid and additional amounts of H_2_O_2_ that scavenge NO, further impairing renal medullary microcirculation. ROS and NO interactions play a crucial role in renal oxygenation and in the generation of CIN. Superoxide radicals, in fact, reduce NO bioavailability through the formation of peroxynitrite, further worsening vasoconstriction but also endothelial dysfunction, since in physiologic conditions NO prevents ROS-mediated endothelial cell injury and reduces transport-dependent ROS formation in the medullary thick ascending limbs (mTALs).

Several experimental studies have shown, either directly [13] or indirectly [14], that administration of CM augments ROS production and renal oxidative stress which, in turn, mediate the damage to cell membranes leading to cellular apoptosis and necrosis, particularly represented in mTALs and in S3 segments of proximal renal tubules of the outer medulla. The injection of CM, in fact, is associated with an increased renal production of ROS metabolites, like malondialdehyde (MDA) or F2 isoprostane, markers of lipid peroxidation; moreover, the administration of ROS scavengers like allopurinol or superoxide desmutase (SOD) was able to prevent the fall in GFR and renal blood flow after CM [15–17]. A consistent rise in superoxide anion content (80%) was also noted in salt-depleted uninephrectomized rats subjected to indomethacin, an experimental model used in rodents to elicit CIN [18].

Clinical studies show similar findings. The urinary excretion of F2 isoprostane was greatly increased in patients undergoing coronary angiography [19] and further enhanced (3-fold increase) in patients with stable chronic renal failure [20]. Urinary xanthine, an end-product in ROS formation, was also increased in patients administered high-osmolar contrast agents, confirming the role of adenosine degradation in generating ROS [21]. Finally, a 2-fold rise in urinary 3-nitrotyrosine, a stable metabolite of peroxynitrite, was recorded immediately after coronary angiography, proportional to the amount of injected CM. This implies that CM administration generates superoxide anions, with subsequent formation of peroxynitrite through a chemical interaction with NO, that inactivate the NO-dependent vasodilatation.

Taken together, both animal and human studies clearly show that ROS generation is enhanced following contrast studies, highlighting their role in the pathogenesis of CIN. Experimental “in vitro” studies, conversely, using LLC-PK1, MDCK, or HEK cells (representing both proximal and distal tubular cells) could not clearly demonstrate a direct role of CM in generating ROS, since in most of these experiments hydrogen peroxide, superoxide anion, or MDA levels were not increased after CM, despite the fact that cellular necrosis and apoptosis were observed, nor could addition of scavengers attenuate the extent of tubular cell damage [22], with the exception of a study in which high concentrations of CM were employed [23]. Recently instead an in vivo and in vitro assessment of pathways involved in CM renal cells apoptosis study of Quintavalle group conclude that CM-induced tubular renal cells apoptosis represents a key mechanism of CIN [24].

The discrepancy with “in vivo” data mostly relies on the different environment in which tubular cells are cultured, far away from that observed in patients.

Nonetheless, cellular studies offer the unique opportunity to evaluate the activation of intracellular signaling pathways involved in cellular apoptosis or necrosis, in the attempt to develop specific therapies to be used in vivo [25]. Recent studies have clarified these aspects either in primary human tubular cells or in HK-2 cells exposed to different types of CM. All the CM determined a decreased cell viability, secondary to a reduced activation of Akt and of ERK 1/2, both playing a pivotal role in cell survival/proliferation, which was substantially alleviated by transfecting the HK-2 cells with a constitutively active form of Akt [26]. In HK-2 cells, it has also been shown that CM affect the activation/deactivation of transcription factors, like FoxO3a and STAT3, which control the genes involved in apoptosis and cell proliferation [27, 28].

## 3. Preventive Strategies on ROS Formation

In these last years, in the attempt to reduce the incidence of CIN, greater attention has been devoted to the use of low- or isoosmolar agents at reduced doses, to the use of specific infusion protocols to hydrate patients, to the elimination of coexisting nephrotoxic agents, and to careful selection of patients. Nevertheless, CIN remains a major adverse iatrogenic complication through the use of CM [2]. Clinical studies conducted over the last decade, contributed to a large extent to the recognition of ROS as major determinants in the pathogenesis of CIN: interestingly, clinical trials aimed to prevent ROS damage in CIN, have preceded experimental studies of CIN models, mostly because some antioxidant agents like N-acetyl-cysteine (NAC) are cheap and harmless in high-risk patients with preexisting renal failure; experimental studies came much later with the attempt to assess the molecular basis of protective mechanisms and to shed light on new therapeutic options.

NAC is a thiol-containing antioxidant able to permeate cell membranes that acts as a cysteine donor for *de novo* cytosolic glutathione synthesis; in vitro studies have shown the ability of NAC to protect in a dose-dependent fashion cultured renal tubular cells incubated with high concentrations of low- and isoosmolar CM [23].

Tepel et al. first reported that administration of NAC was able to drastically reduce the risk of CIN: in a randomized, prospective study 83 patients with chronic stable renal failure undergoing CT scans were enrolled and pretreated with NAC or placebo. The incidence of CIN, defined as a rise in plasma creatinine >0.5 mg/dL within 48 hours, resulted only in 2% of NAC-treated patients, compared to 21% of the placebo group [29].

The efficacy of NAC in CIN prevention was questioned by a number of subsequent clinical trials and meta-analyses showing opposite results [3, 30, 31], suggesting that NAC efficacy could not be clearly proven taking into consideration the patients' heterogeneity of these studies and the impossibility to control several confounding factors, like patients' comorbidities, type of radiologic procedures, CM type and dosage, hydration protocols, doses of NAC, and its timing and mode of administration [32–36]. Nevertheless, given its low toxicity and based on few large, randomized prospective trials, NAC is currently included in prophylactic regimens in high-risk patients with impaired renal function [37–39].

NAC pretreatment is also able to improve renal blood flow, as shown in rats, through a direct renal vasodilation [2] and to attenuate the decline in renal medullary blood flow following CM increasing renal PGE2 and renal cortical NO, but renal parenchymal isoprostane was only marginally influenced [19, 40]; similarly, in patients undergoing coronary angiography, NAC pretreatment did reduce the decline in urinary NO end-products but did not affect lipid peroxidation, evaluated by urinary isoprostane.

This could suggest that NAC enhances eNOS activity and NO generation, but its renal protection could be dissociated from its antioxidant effect.

Bicarbonate infusion has also been proposed as a renoprotective factor in patients undergoing contrast studies; the rationale is that the alkalization might reduce the formation of hydroxyl radicals from hydrogen peroxide and of peroxynitrite. Although the increase of extracellular pH may ameliorate ROS-mediated proximal tubular injury in vitro [40], the impact of bicarbonate on tubular intracellular pH and ROS generation has never been evaluated.

In 119 patients with chronic renal failure, randomized to bicarbonate or saline, Merten et al. have reported a protective effect of sodium bicarbonate infusion on CIN: its solution (1.4%), given as a bolus either 1 hour before CM and for 6 hours after the procedure, was able to attenuate the incidence of CIN compared with the control saline treated patients [41].

In a large prospective study in patients with chronic renal impairment undergoing coronary interventions, the combined administration of NAC and bicarbonate was superior to NAC alone, independent of whether ascorbic acid was present [36]: the incidence of CIN in the former group was 1.9% as compared with 10% in the other group. Other clinical trials with bicarbonate in patients with renal impairment, however, showed conflicting outcomes [42–50].

The results of repeated meta-analyses, however, consistently demonstrate a better outcome with bicarbonate infusion compared to saline-hydration, with a 50% reduction in CIN incidence [51–54], despite the aforementioned heterogeneity of data concerning patients' and procedure-related variables.

Most recently the results of the BOSS trial, which compared sodium bicarbonate and saline for the prevention of a composite of death, renal replacement therapy, or progressive kidney failure over 6 months in patients undergoing any type of angiography, showed no difference on the primary composite endpoint in two group (14.8% in the bicarbonate group versus 16.3% in the saline group, *P* = NS) [55].

A report in patients with chronic renal failure has suggested that the antioxidant probucol may also prevent CIN (8% incidence in probucol-treated patients versus 15%) [56], and few additional studies using ascorbic acid as an antioxidant provided conflicting results [37, 57–59]; a greater number of observations are needed to confirm the efficacy of these substances in preventing CIN.

A further approach to CIN prevention is offered by inhibition of tubular carbonic anhydrase, that results in bicarbonaturia and alkaline urine that might attenuate ROS attack at the apical membrane of tubular epithelium; interestingly, acetazolamide administration ameliorated renal dysfunction in a CIN rat model [60], a result confirmed in children with renal failure undergoing radiocontrast studies and treated with acetazolamide: the rise in urine pH completely prevented the onset of CIN that, conversely, appeared in the 8% of patients treated with bicarbonate [61]; it is noteworthy that acetazolamide prophylaxis is not associated with systemic alkalosis.

To date, however, the real impact of bicarbonate and acetazolamide on tubular intracellular pH and ROS formation during CM administration has not been evaluated.

Data on other drugs effective in reducing CIN potentially by reducing ROS, like statin and ascorbic acid, remain controversial, but recent data suggest that a single high loading dose of atorvastatin administered within 24 hours before CM exposure is effective in reducing the rate of CIN patients at low to medium risk [62].

More recently, our research group evaluated whether a novel isoform of a recombinant Manganese SOD (rMnSOD) could provide an effective protection against CIN: this molecule shares the same ability of physiological SODs in scavenging reactive oxygen species (ROS) but differs from extracellular SODs in that it enters inside the cells after its administration thanks to the presence of a peculiar leader peptide that allows the internalization of the molecule.

We studied the effects of rMnSOD on oxidative damage in a rat model of CIN in uninephrectomized rats; in normal rats, pretreatment with rMnSOD reduced renal superoxide anion production, induced by the activation of NAPDH oxidase, by 84% (*P* < 0.001). In rats treated with high-osmolarity CM, ROS production was almost doubled compared to normal placebo-infused rats (*P* < 0.01) but returned to normal values in rats pretreated with rMnSOD, where a significant increase of SOD activity was detected (+16% versus CM-treated rats, *P* < 0.05). Renal hemodynamics confirmed that the administration of CM determined a striking fall of GFR in CM-treated rats (−70%, *P* < 0.001 versus untreated rats), greatly blunted by rMnSOD administration (−28%, *P* < 0.01); administration of CM was associated with presence of both tubular necrosis and intratubular casts, that were both greatly reduced in SOD-treated rats (both *P* < 0.01). Our conclusions indicate that the scavenging activity of rMnSOD was able to reduce renal oxidative stress and to prevent the reduction of GFR and the renal histologic damage that follows CM administration [63].

In summary, experimental and clinical trials, using NAC, bicarbonate infusion, Probucol, acetazolamide, and rMnSOD seem clearly to suggest that ROS are involved in the pathogenesis of CIN. Nevertheless, whether the efficacy of these interventions are related to ROS antagonism or to the modification of oxygen availability is yet to be defined.

## 4. Conclusions

The pathogenesis of CIN is a paradigm of hypoxic-toxic injury, involving altered renal microcirculation, hypoxia, and ROS-mediated cellular injury. Hypoxic damage develops especially in high-risk patients, in whom renal protective mechanisms, which maintain renal medullary oxygen balance and prevent ROS generation and action, are hampered.

Formation of ROS likely results from the evolving hypoxia and reoxygenation, activating a feed-forward loop of endothelial/vascular dysfunction, upregulation of tubular transport, and the induction of oxygen-consuming reparative mechanisms, with consequently intensified hypoxia. By interfering with hypoxia-adaptive cell responses, ROS might further intensify renal parenchymal injury and dysfunction. Improvement of renal medullary oxygenation and inhibition of ROS formation or ROS scavenging are, therefore, reasonable therapeutic interventions in the prevention of CIN. However, the protective properties attributed to NAC and to bicarbonate infusion or ROS scavenger agents and their putative action through defusing oxidative stress have yet to be established.

## Figures and Tables

**Table 1:** Risk Factors for CIN.

Intrinsic predisposing factors	
Preexisting renal failure	
Diabetes	
Effective blood volume depletion	
Dehydration, hypotension	
Heart failure, cirrhosis, nephrosis	
Hypertension	
Atherosclerosis	
Anemia	
Transplanted kidney	
Aging	
Female gender	
Other nephrotoxins	
Exogenous: nephrotoxic drugs	
Endogenous: heme pigments	
Systemic inflammation	
Extrinsic predisposing factors (procedure related)	
Large CM volume	
Primary coronary intervention/emergency procedure	

## References

[1] Nash K, Hafeez A, Hou S (2002). Hospital-acquired renal insufficiency. *American Journal of Kidney Diseases*.

[2] Heyman SN, Rosen S, Khamaisi M, Idée J-M, Rosenberger C (2010). Reactive oxygen species and the pathogenesis of radiocontrast-induced nephropathy. *Investigative Radiology*.

[3] Kelly AM, Dwamena B, Cronin P, Bernstein SJ, Carlos RC (2008). Meta-analysis: effectiveness of drugs for preventing contrast-induced nephropathy. *Annals of Internal Medicine*.

[4] McCullough PA (2008). Contrast-induced acute kidney injury. *Journal of the American College of Cardiology*.

[5] Rihal CS, Textor SC, Grill DE (2002). Incidence and prognostic importance of acute renal failure after percutaneous coronary intervention. *Circulation*.

[6] Wong PCY, Li Z, Guo J, Zhang A (2012). Pathophysiology of contrast-induced nephropathy. *International Journal of Cardiology*.

[7] Mimic-Oka J, Simic T, Djukanovic L, Reljic Z, Davicevic Z (1999). Alteration in plasma antioxidant capacity in various degrees of chronic renal failure. *Clinical Nephrology*.

[8] Martín-Mateo MC, Sánchez-Portugal M, Iglesias S, de Paula A, Bustamante J (1999). Oxidative stress in chronic renal failure. *Renal Failure*.

[9] Okamura DM, Pennathur S, Pasichnyk K (2009). CD36 regulates oxidative stress and inflammation in hypercholesterolemic CKD. *Journal of the American Society of Nephrology*.

[10] Mehran R, Nikolsky E (2006). Contrast-induced nephropathy: definition, epidemiology, and patients at risk. *Kidney International*.

[11] Lasser EC, Lyon SG, Berry CC (1997). Reports on contrast media reactions: analysis of data from reports to the U.S. Food and Drug Administration. *Radiology*.

[12] Fiaccadori E, Maggiore U, Rotelli C (2004). Plasma and urinary free 3-nitrotyrosine following cardiac angiography procedures with non-ionic radiocontrast media. *Nephrology Dialysis Transplantation*.

[13] Liss P, Nygren A, Erikson U, Ulfendahl HR (1998). Injection of low and iso-osmolar contrast medium decreases oxygen tension in the renal medulla. *Kidney International*.

[14] Rosenberger C, Rosen S, Heyman SN (2006). Renal parenchymal oxygenation and hypoxia adaptation in acute kidney injury. *Clinical and Experimental Pharmacology and Physiology*.

[15] Bakris GL, Lass N, Gaber AO, Jones JD, Burnett JC (1990). Radiocontrast medium-induced declines in renal function: a role for oxygen free radicals. *American Journal of Physiology*.

[16] Toprak O, Cirit M, Tanrisev M (2008). Preventive effect of nebivolol on contrast-induced nephropathy in rats. *Nephrology Dialysis Transplantation*.

[17] Çetin M, Devrim E, Serin Kiliçoğlu S (2008). Ionic high-osmolar contrast medium causes oxidant stress in kidney tissue: partial protective role of ascorbic acid. *Renal Failure*.

[18] Goodman AI, Olszanecki R, Yang LM (2007). Heme oxygenase-1 protects against radiocontrast-induced acute kidney injury by regulating anti-apoptotic proteins. *Kidney International*.

[19] Efrati S, Dishy V, Averbukh M (2003). The effect of N-acetylcysteine on renal function, nitric oxide, and oxidative stress after angiography. *Kidney International*.

[20] Drager LF, Andrade L, Barros de Toledo JF, Laurindo FRM, Machado César LA, Seguro AC (2004). Renal effects of N-acetylcysteine in patients at risk for contrast nephropathy: decrease in oxidant stress-mediated renal tubular injury. *Nephrology Dialysis Transplantation*.

[21] Katholi RE, Woods WT, Taylor GJ (1998). Oxygen free radicals and contrast nephropathy. *American Journal of Kidney Diseases*.

[22] Garofalo AS, Borges FT, Dalboni MA, Santos OFPD (2007). Reactive oxygen species independent cytotoxicity induced by radiocontrast agents in tubular cells (LLC-PK1 and MDCK). *Renal Failure*.

[23] Romano G, Briguori C, Quintavalle C (2008). Contrast agents and renal cell apoptosis. *European Heart Journal*.

[24] Quintavalle C, Brenca M, De Micco F (2011). In vivo and in vitro assessment of pathways involved in contrast media-induced renal cells apoptosis. *Cell Death and Disease*.

[25] Sabbatini M, Santillo M, Pisani A (2006). Inhibition of Ras/ERK1/2 signaling protects against postischemic renal injury. *American Journal of Physiology*.

[26] Andreucci M, Fuiano G, Presta P (2006). Radiocontrast media cause dephosphorylation of Akt and downstream signaling targets in human renal proximal tubular cells. *Biochemical Pharmacology*.

[27] Andreucci M, Faga T, Russo D (2013). Differential activation of signaling pathways by low-osmolar and iso-osmolar radiocontrast agents in human renal proximal tubular cells. *Journal of Cellular Biochemistry*.

[28] Andreucci M, Lucisano G, Faga T (2011). Differential activation of signaling pathways involved in cell death, survival and inflammation by radiocontrast media in human renal proximal tubular cells. *Toxicological Sciences*.

[29] Tepel M, Van Der Giet M, Schwarzfeld C, Laufer U, Liermann D, Zidek W (2000). Prevention of radiographic-contrast-agent-induced reductions in renal function by acetylcysteine. *The New England Journal of Medicine*.

[30] Birck R, Krzossok S, Markowetz F, Schnülle P, Van Der Woude FJ, Braun C (2003). Acetylcysteine for prevention of contrast nephropathy: meta-analysis. *The Lancet*.

[31] Alonso A, Lau J, Jaber BL, Weintraub A, Sarnak MJ (2004). Prevention of radiocontrast nephropathy with N-acetylcysteine in patients with chronic kidney disease: a meta-analysis of randomized, controlled trials. *American Journal of Kidney Diseases*.

[32] Zagler A, Azadpour M, Mercado C, Hennekens CH (2006). N-Acetylcysteine and contrast-induced nephropathy: a meta-analysis of 13 randomized trials. *American Heart Journal*.

[33] Nallamothu BK, Shojania KG, Saint S (2004). Is acetylcysteine effective in preventing contrast-related nephropathy? A meta-analysis. *American Journal of Medicine*.

[34] Pannu N, Manns B, Lee H, Tonelli M (2004). Systematic review of the impact of N-acetylcysteine on contrast nephropathy. *Kidney International*.

[35] Gonzales DA, Norsworthy KJ, Kern SJ (2007). A meta-analysis of N-acetylcysteine in contrast-induced nephrotoxicity: unsupervised clustering to resolve heterogeneity. *BMC Medicine*.

[36] Kshirsagar AV, Poole C, Mottl A (2004). N-acetylcysteine for the prevention of radiocontrast induced nephropathy: a meta-analysis of prospective controlled trials. *Journal of the American Society of Nephrology*.

[37] Briguori C, Airoldi F, D’Andrea D (2007). Renal insufficiency following contrast media administration trial (REMEDIAL): a randomized comparison of 3 preventive strategies. *Circulation*.

[38] Briguori C, Colombo A, Violante A (2004). Standard vs double dose of N-acetylcysteine to prevent contrast agent associated nephrotoxicity. *European Heart Journal*.

[39] Marenzi G, Assanelli E, Marana I (2006). N-acetylcysteine and contrast-induced nephropathy in primary angioplasty. *The New England Journal of Medicine*.

[40] Efrati S, Berman S, Ilgiyeav I, Siman-Tov Y, Averbukh Z, Weissgarten J (2009). Differential effects of N-acetylcysteine, theophylline or bicarbonate on contrast-induced rat renal vasoconstriction. *American Journal of Nephrology*.

[41] Merten GJ, Burgess WP, Gray LV (2004). Prevention of contrast-induced nephropathy with sodium bicarbonate: a randomized controlled trial. *Journal of the American Medical Association*.

[42] Tamura A, Goto Y, Miyamoto K (2009). Efficacy of single-bolus administration of sodium bicarbonate to prevent contrast-induced nephropathy in patients with mild renal insufficiency undergoing an elective coronary procedure. *American Journal of Cardiology*.

[43] Masuda M, Yamada T, Mine T (2007). Comparison of usefulness of sodium bicarbonate versus sodium chloride to prevent contrast-induced nephropathy in patients undergoing an emergent coronary procedure. *American Journal of Cardiology*.

[44] Recio-Mayoral A, Chaparro M, Prado B (2007). The reno-protective effect of hydration with sodium bicarbonate plus N-acetylcysteine in patients undergoing emergency percutaneous coronary intervention: the RENO Study. *Journal of the American College of Cardiology*.

[45] Ozcan EE, Guneri S, Akdeniz B (2007). Sodium bicarbonate, N-acetylcysteine, and saline for prevention of radiocontrast-induced nephropathy. A comparison of 3 regimens for protecting contrast-induced nephropathy in patients undergoing coronary procedures. A single-center prospective controlled trial. *American Heart Journal*.

[46] Pakfetrat M, Nikoo MH, Malekmakan L (2009). A comparison of sodium bicarbonate infusion versus normal saline infusion and its combination with oral acetazolamide for prevention of contrast-induced nephropathy: a randomized, double-blind trial. *International Urology and Nephrology*.

[47] Brar SS, Shen AY-J, Jorgensen MB (2008). Sodium bicarbonate vs sodium chloride for the prevention of contrast medium-induced nephropathy in patients undergoing coronary angiography: a randomized trial. *Journal of the American Medical Association*.

[48] Schmidt P, Pang D, Nykamp D, Knowlton G, Jia H (2007). N-acetylcysteine and sodium bicarbonate versus N-acetylcysteine and standard hydration for the prevention of radiocontrast-induced nephropathy following coronary angiography. *Annals of Pharmacotherapy*.

[49] Adolph E, Holdt-Lehmann B, Chatterjee T (2008). Renal insufficiency following radiocontrast exposure trial (REINFORCE): a randomized comparison of sodium bicarbonate versus sodium chloride hydration for the prevention of contrast-induced nephropathy. *Coronary Artery Disease*.

[50] Maioli M, Toso A, Leoncini M (2008). Sodium bicarbonate versus saline for the prevention of contrast-induced nephropathy in patients with renal dysfunction undergoing coronary angiography or intervention. *Journal of the American College of Cardiology*.

[51] Hogan SE, L’Allier P, Chetcuti S (2008). Current role of sodium bicarbonate-based preprocedural hydration for the prevention of contrast-induced acute kidney injury: a meta-analysis. *American Heart Journal*.

[52] Navaneethan SD, Singh S, Appasamy S, Wing RE, Sehgal AR (2009). Sodium bicarbonate therapy for prevention of contrast-induced nephropathy: a systematic review and meta-analysis. *American Journal of Kidney Diseases*.

[53] Meier P, Ko DT, Tamura A, Tamhane U, Gurm HS (2009). Sodium bicarbonate-based hydration prevents contrast-induced nephropathy: a meta-analysis. *BMC Medicine*.

[54] Ho KM, Morgan DJ (2008). Use of isotonic sodium bicarbonate to prevent radiocontrast nephropathy in patients with mild pre-existing renal impairment: a meta-analysis. *Anaesthesia and Intensive Care*.

[55] Solomon R

[56] Li G, Yin L, Liu T (2009). Role of probucol in preventing contrast-induced acute kidney injury after coronary interventional procedure. *American Journal of Cardiology*.

[57] Jo S-H, Koo B-K, Park J-S (2009). N-acetylcysteine versus AScorbic acid for preventing contrast-induced nephropathy in patients with renal insufficiency undergoing coronary angiography NASPI study-a prospective randomized controlled trial. *American Heart Journal*.

[58] Spargias K, Alexopoulos E, Kyrzopoulos S (2004). Ascorbic acid prevents contrast-mediated nephropathy in patients with renal dysfunction undergoing coronary angiography or intervention. *Circulation*.

[59] Boscheri A, Weinbrenner C, Botzek B, Reynen K, Kuhlisch E, Strasser RH (2007). Failure of ascorbic acid to prevent contrast-media induced nephropathy in patients with renal dysfunction. *Clinical Nephrology*.

[60] Hellman RN, Novikov M, Campos S (2004). Acetazolamide is renoprotective in a rat model of high dose contrast media induced renal failure. *Journal of the American Society of Nephrology*.

[61] Assadi F (2006). Acetazolamide for prevention of contrast-induced nephropathy: a new use for an old drug. *Pediatric Cardiology*.

[62] Quintavalle C, Fiore D, De Micco F (2012). Impact of a high loading dose of atorvastatin on contrast-induced acute kidney injury. *Circulation*.

[63] Pisani A, Sabbatini M, Riccio E (2013). Effect of a recombinant manganese superoxide dismutase on prevention of contrast-induced acute kidney injury. *Clinical and Experimental Nephrology*.

